# Gonadotrophin-Releasing Hormone (GnRH) Analogues in the Treatment of Mixed Mullerian Tumours of the Uterus: Two Case Reports and Review

**DOI:** 10.1080/13577149877975

**Published:** 1998-12

**Authors:** Michael Katesmark, Frank Lawton

**Affiliations:** Department of Obstetrics & Gynaecology King's College Hospital Denmark Hill London SE5 9RS UK

## Abstract

*Subjects/Discussion.* Two cases of clinical and radiological response of recurrent mixed Mullerian tumours following treatment with either nasal (Buserilin) or intramuscular (Goserilin) GnRH analogues are reported and a short review of the evidence to support this treatment option presented.


*Sarcoma* (1998) **2**, 197–200

SHORT REPORT

### Gonadotrophin-releasing hormone (GnRH) analogues in the treatment of mixed Mullerian tumours of the uterus: two case reports and review

MICHAEL KATESMARK & FRANK LAWTON

*Department of Obstetrics & Gynaecology, King's College Hospital, London SE5 9RS, UK*

**Abstract**

*Subjects/Discussion.* Two cases of clinical and radiological response of recurrent mixed Mullerian tumours following treatment with either nasal (Buserilin) or intramuscular (Goserilin) GnRH analogues are reported and a short review of the evidence to support this treatment option presented.

**Key words**: *mixed Mullerian uterine tumours, GnRH analogues.*

### Introduction

Mixed Mullerian tumours, derived from the mesenchymal remnants of the urogenital ridge and epithelium originating from the coelomic cavity, are rare but usually rapidly fatal uterine tumours of post-reproductive women. Recurrence after surgery is common even in apparently early disease and survival despite subsequent radiotherapy or chemotherapy extremely poor.

The *in vitro* finding of GnRH receptors on some tumour lines led us to try treatment with nasal GnRH analogues in two patients with some significant, albeit temporary, success.

### Case 1

A 70 year-old mother of eight children, presented in April 1993 with a short history of post menopausal-bleeding. She had passed through the menopause aged 38 and had never taken hormone replacement therapy. Her past medical history included recently diagnosed non-insulin dependent diabetes mellitus with nothing else of relevance.

There was no evidence of uterine enlargement on examination, but Vabra curettage suggested a heterologous malignant mixed Mullerian tumour (Carcinosarcoma) and she subsequently underwent a total abdominal hysterectomy with bilateral salpingo-oophorectomy and omentectomy. There was no evidence of extra-uterine spread at laparotomy and histology confirmed a poorly differentiated carcinosarcoma with no myometrial invasion (Stage 1a).

Despite the early stage at presentation, within a year (March 1994) she developed pelvic pain associated with a palpable vaginal mass. A CT scan confirmed a soft tissue mass 4 × 5 cm above the vaginal vault causing partial right ureteric obstruc-

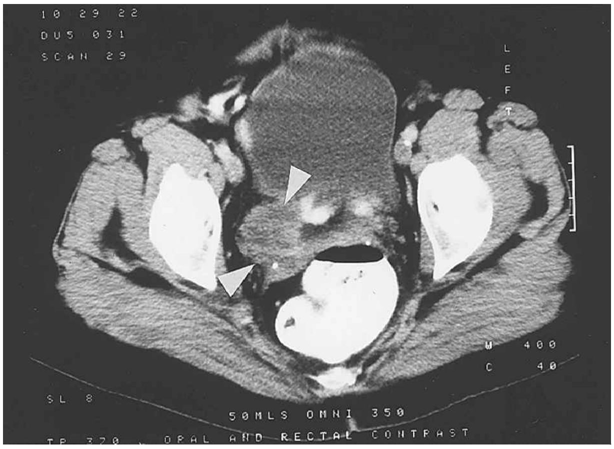

**Fig. 1.** *CT scan (pelvis) of patient (Case 1) before treatment with a GnRH analogue demonstrating a right-sided mass of recurrent tumour (arrowed) causing ureteric obstruction.*

Correspondence to: M. Katesmark, Department of Obstetrics & Gynaecology, King's College Hospital, Denmark Hill, London SE5 9RS, UK.

The authors would welcome enquires from researchers able to offer reliable means of demonstrating GnRH receptors on pathological specimens obtained in the above cases.

1357-714X/98/030197-04 $9.00 © 1998 Carfax Publishing Ltd

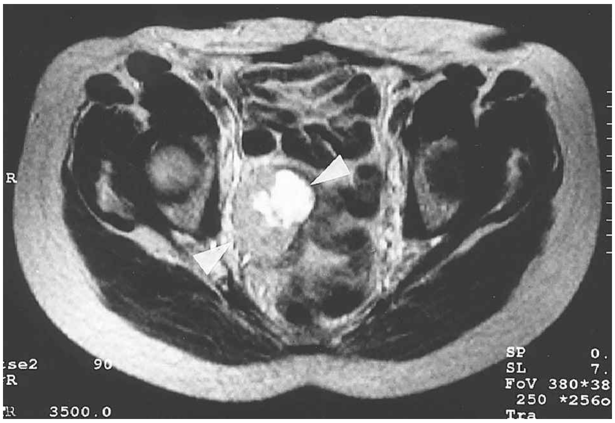

**Fig. 2.** *MRI scan (pelvis) of patient (Case 1) three months after commencing treatment with a GnRH analogue demonstrating central necrosis of tumour with no significant enlargement (arrowed).*

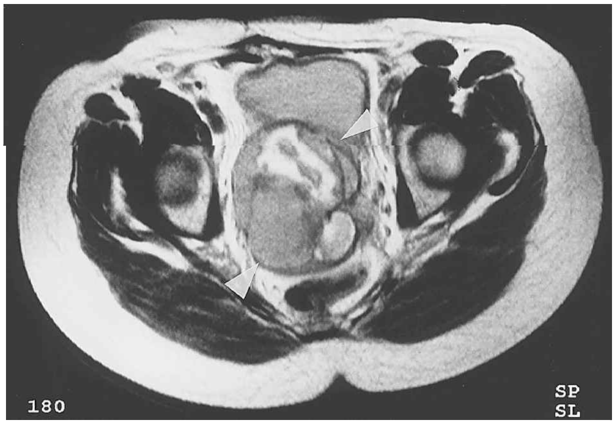

**Fig. 3.** *MRI scan (pelvis) of patient (Case 1) three months after completing treatment with a GnRH analogue. Post-treatment evidence of enlargement is now present (arrowed) but areas of central necrosis persist and there is compression rather than invasion of local structures.*

tion and right-sided pelvic lymphadenopathy extending to the para caval region (Fig. 1).

In view of her pain and the rapid return of disease she was commenced on Goserelin acetate 3.6 mg/28 days (Zoladex, ICI, UK) with rapid resolution of symptoms. Pelvic examination was normal in June 1994 and magnetic resonance imaging (MRI) performed the same month showed no significant increase in tumour size or progression of the pelvic lymphadenopathy (Fig. 2).

She remained in both radiological and clinical remission and completed the six-month course of treatment. Further MRI assessment in December 1994 showed evidence of central necrosis in the tumour mass and no lymphadenopathy (Fig. 3).

In March 1995 a further episode of vaginal bleeding heralded the return of progressive tumour. An offensive polypoid tumour arising from the vaginal vault was excised and combined radiotherapy and nasal Buserelin acetate 150 $\mu$g TDS (Suprecur, Hoeschst, UK) suppressed her disease until early 1996. From February 1996 there was rapid local growth in the pelvis and she died from obstructive renal failure in April 1996.

**Case 2**

A 49 year-old mother of two was referred from a menopause clinic with a short history of irregular vaginal bleeding on combined hormone replacement therapy, which she had taken for five years. She had a complex past medical history featuring several operations for adenocarcinoma of the bowel (complicating polyposis coli), culminating in a panproctocolectomy with ileostomy formation in 1986. A further operation for bowel obstruction had taken place in 1989, with no evidence of tumour recurrence.

Vabra curettings obtained on 5 July 1993 demonstrated apparently poorly differentiated adenocarcinoma of endometrial origin, but immunocytochemistry of the subsequent hysterectomy specimen confirmed a heterologous malignant mixed mesodermal tumour. Tumour volume was small and infiltration confined to the superficial myometrium only (Stage 1b).

After some initial complications arising from her previous extensive pelvic surgery, she remained well until December 1994, when she developed pelvic pain and haematuria associated with a pelvic mass. MRI scanning confirmed a $5 \times 5$ cm vault mass extending to S3, with no pelvic lymphadenopathy. Subsequent cystoscopy excluded invasive tumour in the bladder.

She was commenced on Goserelin acetate 3.6 mg/28 days in February 1994. There was rapid clinical regression, and both a CT scan and MRI performed in June 1994 showed no evidence of tumour progression.

After completing six cycles of Goserilin she remained in remission until November 1994, when increasing pelvic and low back pain was found to be associated with radiological recurrence. She was commenced on salvage radiotherapy (Mid plain dose of 40 Gy in 20 daily fractions); despite some initial success in symptom reduction, she developed recurrent small bowel obstruction associated with progressive left iliac lymphadenopathy. The former was thought more likely to be due to surgical adhesions rather than tumour, and with careful dietary adjustment and pain control she remained reasonably well until April 1996.

Increasing back pain was associated with tumour re-growth and was treated with three cycles of Cyclophosphamide (Farmitalia, UK) and Cisplatin (Farmitalia, UK) in early 1996; clinically there was a partial response, but radiological confirmation was difficult because of her previous surgery. Incontrovertible evidence of tumour progression was, however, shown on PET scanning in March 1997, and she is currently managed on MST 300 mg bd,

**Table 1**. *Classification of mixed Mullerian tumours*

| Mesenchymal component | Epithelial component: Benign | Epithelial component: Malignant | Tumour type |
|---|---|---|---|
| Benign | Adenofibroma | Adenosarcoma | Homologous |
| | Undescribed | Adenosarcoma | Heterologous |
| Malignant | Carcinofibroma | Carcinosarcoma | Homologous |
| | Carcinomesenchymoma | Carcinosarcoma | Heterologous |

Amitriptyline 10 mg nocte and Oromorph 40 mg as required.

### Discussion

Mixed Mullerian tumours are traditionally divided into two groups depending on whether the mesenchymal and epithelial elements are uterine ('homologous', e.g. smooth muscle, endometrial stroma) or non-uterine ('heterologous' e.g. striated muscle, cartilage, bone) in origin (Table 1). Carcinosarcomas, containing malignant components from both cell lines, are commoner than the pure uterine sarcomas but still comprise less than 2% of uterine tumours.[1,2] They are rare during reproductive life, with a median incidence at age 65.[3]

Risk factors overlap with endometrial cancer (namely hypertension, diabetes and nulliparity) but to a lesser degree.[2] The most reliable risk factor is previous pelvic irradiation, although the true incidence (between 5 and 35% at ten years) is disputed.[4]

Staged in the same manner as endometrial tumours, treatment of early disease is by total abdominal hvsterectomy and bilateral salpingo-oophorectomy with peritoneal cytological sampling.[5] The role of lymphadenectomy remains unclear. Prognostic indicators such as the degree of mitotic activity, cellular atypia and cervical involvement are less useful than in endometrial tumours,[6] although vascular involvement and positive cytology are of sinister portent.[7]

Whether to treat with adjuvant radiotherapy or chemotherapy (and indeed the optimum timing and/or agent used) is still uncertain, with distant recurrence a perpetual problem.[2] Advanced (stage III and IV) or recurrent disease has an appalling prognosis regardless of treatment.[8]

Specific GnRH receptors have been demonstrated in normal myometrium, leiyomyomata,[9] epithelial ovarian and endometrial cancer cells,[10–12] and a number of human cancer cell lines (MCF-7, MDA-MB-231, LNCaP).[13] *In vitro* inhibition of growth by GnRH analogues has been clearly demonstrated in ovarian tumours[14] and there has been some early (and limited) success in treating advanced endometrial and ovarian cancer with Goserelin.[15,16]

In the above cases the clinical situation was judged to warrant intervention with as few side-effects as possible. Both women were infirm and reluctant to undergo radiotherapy or aggressive chemotherapy; adverse effects of GnRH treatment were thought to be unlikely and treatment commenced on the first Hippocratic principle.

There was confirmed clinical remission in both cases lasting over a year with no significant side-effects. We suggest that further research in elucidating the role of GnRH analogues in the treatment of these rare tumours is indicated at both a cellular and therapeutic level.

### References

1 Salazar OM, Bonfiglio TA, Patten SF. Uterine sarcomas: natural history, treatment and prognosis. *Cancer* 1978; 42:1152.

2 Olah KS, Gee H, Blunt S, Dunn JA, Kelly K, Chan KK. Retrospective analysis of 318 cases of uterine sarcoma. *Eur J Cancer* 1991; 27:1095.

3 Lurain JR, Piver MS. Uterine sarcomas: clinical features and management. In: Copplestone M, Ed. *Gynecologic Oncology*, Vol II, 2nd edition. Edinburgh: Churchill Livingstone, 1992:827.

4 Rodriguez J, Hart W. Endometrial tumours occurring 10 or more years after pelvic irradiation for carcinoma. *Int J Gynaecol Pathol* 1982; 1:125.

5 Doss LL, Llorens AS, Henriquez EM. Carcinosarcoma of the uterus: a forty-year experience from the state of Missouri. *Gynecol Oncol* 1984; 18:43–48.

6 Marth C, Koza A, Muller-Holzner E, Hetzel H, Fuith LC, Dapunt O. Prognostically relevant factors in malignant mixed Mullerian tumors of the uterus. *Geburtshilfe Frauenheilk* 1990; 50:605.

7 Schweizer W, Demopoulos R, Beller U, Dubin N. Prognostic factors for malignant mixed mesodermal tumours of the uterus. *Int J Gynaecol Pathol* 1990; 9:129–33.

8 Chiara S, Foglia G, Odicini F. Uterine sarcomas: a clinicopathological study. *Oncology* 1988; 45:428–34.

9 Chegini N, Rong H, Dou Q, Kipersztok S, Williams RS. Gonadotrophin-releasing hormone and GnRH receptor gene expression in human myometrium and leiomyomata. *J Clin Endocrinol Metab* 1996; 81:3215–21.

10 Emons G, Schroder B, Ortmann O, Westphalen S, Schultz KD, Schally AV. High affinity binding and direct antiproliferative effects of luteinising hormone-releasing analogues in human endometrial cancer cell lines. *J Clin Endocrinol Metab* 1993; 77(6):1458–64.

11 Takagi H, Imai A, Furui T, Horibe S, Fuseya T, Tamaya T. Evidence for tight coupling of gonadotrophin-releasing hormone receptors to phoshatidylinositol kinase in plasma membrane from gynaecological carcinomas. *Gynecol Oncol* 1995; 58(1):110–5.
12 Wade K, Quinn MA, Hammond I, Williams K, Cauchi M. Uterine sarcoma: steroid receptors and response to hormonal therapy. *Gynecol Oncol* 1990; 39:364.
13 Palvi I, Vincze B, Kalnay A, Turi G, Mezo I, Teplan I, Seprodi J. Effect of gonadotrophin-releasing hormone analogues and their conjugates on gonadotrophin-releasing hormone receptor-positive human cancer cell lines. *Cancer Detect Prev* 1996; 20(2):146–52.
14 Connor JP, Buller RE, Conn PM. Effects of GnRH analogues on six ovarian cancer cell lines in culture. *Gynecol Oncol* 1994; 54(1):80–6.
15 Van der Vange N, Greggi S, Burger CW, Kenemans P, Vermorken JB. Experience with hormone therapy in advanced epithelial ovarian cancer. *Acta Oncol* 1995; 34(6):813–20.
16 de Vriese G, Bone J. Possible role of goserilin, an LH-RH agonist in the treatment of gynaecological cancers. *Eur J Gynaecol Oncol* 1993; 14(3):187–91.
